# Factors associated with emotional regulation self-efficacy in adolescents hospitalized for intentional drug and chemical overdose: a cross-sectional study

**DOI:** 10.3389/fpsyt.2026.1793066

**Published:** 2026-05-22

**Authors:** Ting Li, Nengyue Chen, Shaohua Zhang, Shaodong Zhao, Li Zhao

**Affiliations:** 1 Department of Emergency, Children’s Hospital of Nanjing Medical University, Nanjing, Jiangsu, China; 2 Health Management Center, Children’s Hospital of Nanjing Medical University, Nanjing, Jiangsu, China

**Keywords:** adolescent self-harm, depressive symptoms, family functioning, regulatory emotional self-efficacy, social support

## Abstract

**Background:**

Intentional drug and chemical overdose (IDCO) represents a frequent, high-risk form of self-harm among adolescents, often involving accessible substances and carrying significant medical and psychological sequelae. Regulatory emotional self-efficacy (RESE), a core indicator of an individual’s capacity for emotional management, is posited to critically influence both the motivation for and the clinical trajectory following IDCO. However, research specifically examining RESE within this distinct, high-acuity IDCO population remains scarce, limiting the development of targeted psychosocial interventions.

**Objective:**

This study aimed to explore the correlations between RESE and depressive symptoms, social support, family functioning, and clinical indicators, and to identify factors associated with RESE. As a secondary exploratory aim, we examined factors associated with clinical severity.

**Methods:**

A total of 145 adolescents aged 10–13 years hospitalized for Intentional drug and chemical overdose between July 2023 and June 2025 were enrolled. Participants were stratified by sex into female (n=99) and male (n=46) groups, and by drug category into psychotropic, neurological/antiepileptic, antibiotic/cold remedy, pesticide/household chemical, and other groups. All participants completed the Regulatory Emotional Self-Efficacy Scale, Depressive Symptom Scale, Social Support Scale, and Family Functioning Scale.

**Results:**

Regulatory emotional self-efficacy scores were significantly lower in females than in males (t=3.637, P = 0.001). Significant differences in self-efficacy scores were observed across drug categories (F = 3.456, P = 0.009), with the pesticide/household chemical group scoring the lowest. Regulatory emotional self-efficacy was negatively correlated with depressive symptoms (r=-0.47, P<0.001), positively correlated with social support (r=0.50, P<0.001). Multiple linear regression identified depressive symptoms (t=-3.372, P = 0.001), social support (t=3.876, P<0.001), and family functioning (t=-3.423, P = 0.001) as factors independently associated with regulatory emotional self-efficacy. In secondary exploratory analyzes, clinical severity was positively associated with depressive symptoms (OR = 1.578, 95% CI: 1.234-2.019, P<0.001), and pesticide/household chemical exposure was associated with higher odds of moderate-to-severe clinical presentation (OR = 3.434, 95% CI: 1.401-8.417, P = 0.007).

**Conclusion:**

RESE shows significant independent associations with depressive symptoms, social support, and family functioning in adolescents hospitalized for Intentional drug and chemical overdose. These cross-sectional findings identify potential targets for future prospective intervention research but do not directly support claims of improved management efficacy.

## 1 Introduction

Intentional drug and chemical overdose (IDCO) among adolescents represents a prevalent form of non-suicidal self-injury (NSSI), strongly linked to emotional dysregulation and impairments in impulse control (1, 2). Regulatory Emotional Self-Efficacy (RESE), a central construct in social cognitive theory, reflects an individual’s perceived capability to manage emotions and directly shapes behavioral decisions under stress (3, 4). Evidence indicates that adolescents with diminished RESE levels are more susceptible to internalizing symptoms, including depression and anxiety, as well as impulsive self-harm behaviors (5, 6). Within IDCO populations, RESE may modulate the severity and recurrence of overdose incidents by balancing negative emotional responses and behavioral inhibition. Moreover, social support systems and family functioning serve as protective factors for adolescent resilience, where heightened family cohesion and social resources mitigate the adverse psychological impacts of stressful events (7).

Although RESE’s role in adolescent emotional and behavioral issues has gained increasing attention, research specific to high-risk IDCO groups remains notably scarce. Existing studies predominantly focus on RESE profiles in individuals with depression or anxiety disorders in community or outpatient settings (5, 6), lacking dedicated investigations into adolescent IDCO cohorts who present with acute medical crises and may possess unique psychosocial profiles related to substance accessibility, intent ambiguity, and post-overdose psychological responses (8). Furthermore, most research fails to account for potential variations in psychological and behavioral traits across drug types, wherein the use of highly toxic agents (e.g., pesticides and household chemicals) may signal more acute psychological crises, necessitating deeper inquiry into their relationship with RESE (9). Methodologically, prior work often relies on single time-point cross-sectional data, overlooking dynamic interactions between clinical severity and psychological variables (10, 11). Inadequate integration of standardized assessment tools further constrains the translational applicability of findings for clinical interventions (12, 13). This gap hinders the translation of RESE theory into effective emergency and follow-up care for this vulnerable group.

This cross-sectional study employs standardized instruments—the Regulatory Emotional Self-Efficacy Scale, Depression Symptom Scale, Social Support Scale, and Family Functioning Scale-to systematically gather psychosocial and clinical data from adolescents with IDCO. Unlike previous research, the present sample encompasses subgroups based on drug type (e.g., psychotropic agents, neuro/antiepileptic drugs, antibiotics/antiviral medications, pesticides/household chemicals, and others), enabling analysis of associations between drug selection behaviors and RESE (14, 15). Multiple linear regression models, adjusted for confounders such as gender and age, will be used to examine independent associations between depressive symptoms, social support, family functioning, and RESE. Concurrently, as a secondary exploratory analysis, logistic regression will be employed to explore cross-sectional correlates of clinical severity. This study aims to address critical gaps in RESE research among adolescents with IDCO and provide empirical foundations for developing tailored psychosocial interventions.

## 2 Materials and methods

This study was conducted at the Children’s Hospital of Nanjing Medical University, a tertiary care academic medical center and the largest pediatric referral hospital in Jiangsu Province, China. The hospital serves a catchment area of approximately 10 million people, with approximately 200,000 emergency department visits annually. The Department of Emergency manages approximately 300–400 cases of pediatric poisoning each year, including both accidental and intentional exposures. The hospital is equipped with a dedicated Pediatric Intensive Care Unit (PICU), psychiatric consultation services, and a multidisciplinary team for managing self-harm in adolescents. Data collection occurred between July 2023 and June 2025. All procedures adhered to the principles of the Declaration of Helsinki. The study protocol was reviewed and approved by the Institutional Ethics Committee of Children’s Hospital of Nanjing Medical University (202302026-1). This study was conducted at our institution from July 2023 to June 2025. All procedures adhered to the principles of the Declaration of Helsinki and were approved by the Institutional Ethics Committee. Written informed consent was obtained from all participants and/or their legal guardians.

### 2.1 General information

In this cross-sectional study, a total of 145 consecutive adolescents aged 10–13 years hospitalized for Intentional drug and chemical overdose between July 2023 and June 2025 were enrolled, including 99 females (68.28%) and 46 males (31.72%). Age distribution was as follows: 43 participants (29.66%) aged 10–11 years, 51 (35.17%) aged 11–12 years, and 51 (35.17%) aged 12–13 years, with a mean age of 11.82 ± 1.03 years (range: 10.0–13.9 years; i.e., all participants were younger than 14 years of age). Sample size calculation was performed using G*Power 3.1 software for multiple linear regression analysis. Based on an anticipated medium effect size (f² = 0.15), α = 0.05, and power (1–β) = 0.80, the initial calculation with 7 planned predictors indicated a minimum required sample size of 103. Following bivariate screening and theoretical considerations, the final regression model included 11 predictors. We therefore recalculated the required sample size for 11 predictors, which yielded a minimum requirement of 123 participants. Our final enrollment of 145 participants exceeds this threshold, achieving a *post hoc* statistical power of 0.86, which remains adequate for detecting medium effects in the expanded model.

### 2.2 Inclusion and exclusion criteria

#### 2.2.1 Inclusion criteria

(1) Age 10 to 13 years (i.e., ≥10.0 and <14.0 years), confirmed by legal guardians;(2) Clinically diagnosed with intentional self-harm behavior (including both non-suicidal self-injury and suicide attempts) by an attending psychiatrist based on comprehensive clinical evaluation at the time of admission ;(3) Time from drug ingestion to hospital presentation <12 hours;(4) Signed informed consent from both patients and guardians.

#### 2.2.2 Exclusion criteria

(1) Severe impairment of consciousness (Glasgow Coma Scale score <12) or unstable vital signs; (2) Comorbid severe physical diseases (e.g., cardiac, hepatic, or renal failure); (3) Diagnosis of intellectual disability or pervasive developmental disorder; (4) Refusal to participate or withdrawal during the study.

### 2.3 Methods

#### 2.3.1 Study design

A hospital-based, cross-sectional study design was employed. A consecutive sampling method was used to enroll all eligible adolescents presenting with Intentional drug and chemical overdose during the study period.

#### 2.3.2 Data collection process

Data collection commenced with the extraction of demographic and clinical information from the Hospital Information System (HIS). This was followed by standardized scale assessments. All raters completed a two-week standardized training program, achieving an inter-rater reliability with a kappa coefficient exceeding 0.85.

#### 2.3.3 Quality control

To ensure data integrity, a dual-independent data entry procedure was adopted. A random selection of 10% of cases underwent verification, yielding a data consistency rate of 98.5%. Weekly data quality control meetings were held to maintain standardization throughout the data acquisition process.

#### 2.3.4 Operational definition of intentional drug and chemical overdose

Intentional drug and chemical overdose was defined as the Intentional self-administration of a medication or chemical substance in excess of the prescribed or recommended dosage, with the expressed or inferred intent of causing self-harm. The determination of intentionality was made through a multi-step process: (1) initial triage assessment by emergency department nurses using standardized questions about the circumstances of ingestion; (2) separate clinical interviews conducted by both the attending emergency physician and a consulting psychiatrist; (3) review of collateral information from family members or witnesses when available; and (4) final confirmation by the attending psychiatrist. Cases were classified as accidental ingestion if there was clear evidence of unintentional exposure (e.g., medication errors by caregivers, exploratory ingestion by younger children without evidence of self-harm intent) or if the patient and family consistently denied self-harm intent and no behavioral indicators of intentionality were present. This determination was completed within 24 hours of admission, prior to enrollment in the study.

#### 2.3.5 Strategies to minimize bias

Several strategies were implemented to minimize potential bias in this study:

##### 2.3.5.1 Selection bias

Consecutive sampling of all eligible patients during the study period minimized selection bias. Explicit inclusion and exclusion criteria were applied uniformly by trained research staff.

##### 2.3.5.2 Information bias

Standardized data collection forms and protocols were used for all participants. All raters completed a two-week standardized training program, achieving inter-rater reliability with a kappa coefficient > 0.85. Scales were administered in a private setting to minimize social desirability bias, and participants were assured of confidentiality.

##### 2.3.5.3 Measurement bias

Validated instruments with established psychometric properties in Chinese adolescent populations were used. The Poisoning Severity Score was independently determined by two attending physicians, with disagreements resolved by a third senior consultant.

##### 2.3.5.4 Recall bias

To minimize recall bias, clinical data (e.g., rescue time, length of stay) were extracted from the Hospital Information System rather than relying on participant recall. Psychological scales were administered as soon as clinically feasible after stabilization (typically within 24–48 hours of admission) to reduce recall error regarding pre-admission psychological states.

##### 2.3.5.5 Data management bias

Dual-independent data entry with verification of 10% randomly selected cases (98.5% consistency rate) minimized data entry errors. Weekly data quality control meetings ensured ongoing adherence to protocols.

### 2.4 Outcome measures

#### 2.4.1 Regulatory emotional self-efficacy

The Regulatory Emotional Self-Efficacy Scale (RESE), was employed. Items are rated on a 5-point Likert scale (1=not confident at all, 5=very confident), covering two dimensions: expressing positive emotions (6 items) and managing despondency/distress (10 items). Total scores range from 16 to 80, with higher scores indicating greater self-efficacy in emotion regulation. The Chinese version of the RESE has been validated in early adolescents, demonstrating good construct validity and test-retest reliability. In this study, the scale demonstrated a Cronbach’s alpha of 0.87.

#### 2.4.2 Depressive symptoms

Depressive symptoms were evaluated using the 9-item Patient Health Questionnaire (PHQ-9). Responses are graded on a 4-point scale (0=not at all, 3=nearly every day), yielding a total score between 0 and 27. A score ≥ 10 suggests the presence of clinically relevant depressive symptoms. The Chinese version of the PHQ-9 has been validated in adolescent populations, showing good sensitivity and specificity for detecting depressive disorders. The internal consistency, measured by Cronbach’s alpha, was 0.83 in our cohort.

#### 2.4.3 Clinical severity

The Poisoning Severity Score (PSS) was utilized to grade clinical severity into four levels: 0 (asymptomatic), 1 (mild), 2 (moderate), and 3 (severe). The PSS has been widely used in poisoning research internationally; however, specific validation studies in Chinese adolescent populations are limited. The scale’s utility in this context relies on its objective clinical criteria. PSS ratings were independently determined by two attending physicians. Discrepancies were resolved through adjudication by a third senior consultant.

#### 2.4.4 Rescue time

This was defined as the time interval from the initiation of rescue procedures to the point of clinical stabilization, defined as stable vital signs maintained for 24 consecutive hours. This duration was recorded in hours.

#### 2.4.5 Length of stay

The LOS was defined as the total duration (in days) from the time of emergency department registration to the time of discharge from the emergency observation unit, general inpatient ward, or PICU. Methodological note: Because LOS encompasses heterogeneous care pathways (e.g., short-stay observation vs. PICU admission), it should be interpreted as an operational descriptor of resource utilization rather than a direct measure of physiological recovery. Comparisons of LOS across patients with different disposition pathways warrant caution.

#### 2.4.6 Drug type

Ingested substances were categorized according to the World Health Organization’s Anatomical Therapeutic Chemical (ATC) Classification System. Primary categories included psychotropic agents (ATC code N), nervous system drugs (ATC code N), and antibacterials for systemic use (ATC code J). Specific classifications analyzed were: Psychotropic Drugs, Neurological/Antiepileptic Drugs, Antibiotics/Anti-cold Medications, Pesticides/Household Chemicals, and Others.

#### 2.4.7 Level of social support

Social support was measured using the Adolescent Social Support Scale (ASSS). This 12-item tool uses a 4-point scoring system, producing total scores from 12 to 48. Higher scores are indicative of more robust perceived social support. The ASSS was developed and validated in Chinese adolescent populations, demonstrating good construct validity and internal consistency. The scale’s Cronbach’s alpha coefficient was 0.79 in the present investigation.

#### 2.4.8 Family functioning

Family functioning was assessed using the 12-item General Functioning subscale of the Family Assessment Device (FAD), scored on a 4-point Likert scale with a total score ranging from 12 to 48. Higher scores indicated poorer family functioning. The Chinese version of the FAD has been validated in adolescent and family studies, showing good factorial validity and reliability. In the present study, the Cronbach’s α for this subscale was 0.81.

#### 2.4.9 Assessment of self-harm and suicidality

In this study, ‘intentional self-harm’ was operationalized as an Intentional act of Intentional drug and chemical overdose with the stated intent of causing harm to oneself, as documented in the medical record by the attending physician following clinical interview. Consistent with standard clinical practice at our institution, the distinction between non-suicidal self-injury (NSSI) and suicidal behavior disorder was made clinically but not systematically captured using structured research instruments for this study. The PHQ-9 includes an item on thoughts of self-harm; however, a detailed assessment of suicidal intent severity (e.g., using the Beck Scale for Suicide Ideation) was not performed, representing an important direction for future research.

### 2.5 Statistical analysis

Statistical analyzes were performed using SPSS version 26.0 (IBM Corporation, USA). Normality of continuous variables was assessed using the Shapiro–Wilk test. Normally distributed variables were expressed as mean ± standard deviation ( $\bar{x}$ ± SD), while non-normally distributed variables were summarized as median (interquartile range) [M (Q1, Q3)]. Categorical variables were described as frequency (percentage). Correlation analyzes between observational indicators and emotion regulation self-efficacy were conducted using Pearson or Spearman correlation coefficients, as appropriate. Multiple linear regression was employed to identify factors influencing emotion regulation self-efficacy, incorporating variables such as age, sex, type of drug ingested, and PHQ-9 score. Multicollinearity was assessed using variance inflation factor (VIF), with VIF < 5 indicating no significant multicollinearity. All tests were two-tailed, and a p-value < 0.05 was considered statistically significant.

For the secondary exploratory multivariable logistic regression analysis examining factors associated with clinical severity, variable selection was guided by both theoretical considerations and bivariate screening results. These analyzes are presented as hypothesis-generating rather than confirmatory. Candidate variables included all demographic, clinical, and psychosocial factors assessed in the study. Variables were retained in the final model if they met either of the following criteria: (1) theoretical relevance based on prior literature (e.g., agent type, depressive symptoms) or (2) association with clinical severity in bivariate analyzes at P < 0.10. We acknowledge that for rescue time, the direction of association cannot be determined from cross-sectional data; longer rescue time may reflect more severe initial poisoning rather than being an independent risk factor. Social support and family functioning scores were not included in the final logistic regression model because they showed no significant association with clinical severity in bivariate analyzes and were not identified as direct predictors of clinical severity in theoretical frameworks. However, potential theoretical pathways involving depressive symptoms and RESE are noted in the discussion as avenues for future hypothesis testing.

## 3 Results

### 3.1 Baseline characteristics of patients

**Figure 1:**
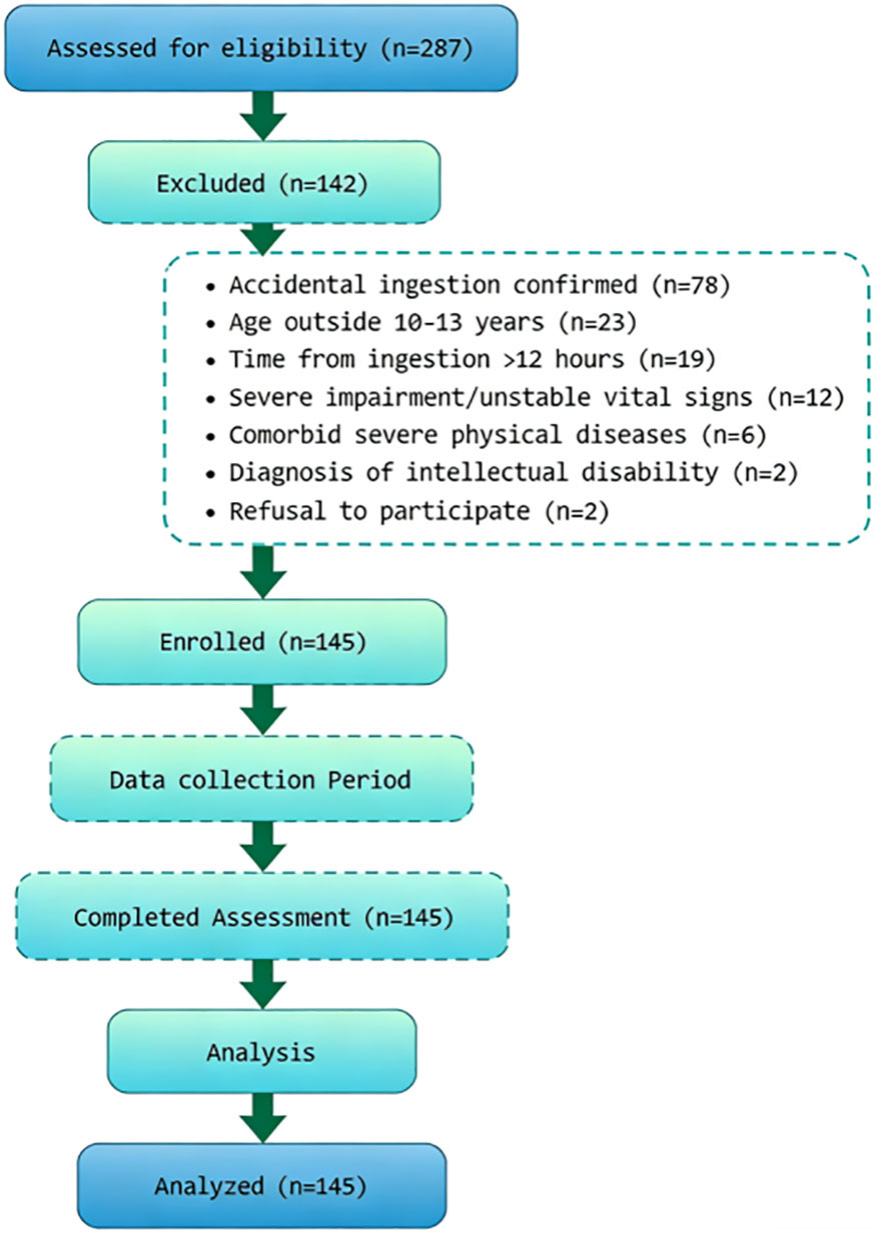
The participant flow is illustrated.

Patient age was presented as mean ± SD. Females constituted the majority of the cohort. Psychotropic agents were the most common type of drug involved. Other categories included neuroactive/antiepileptic drugs, antibiotics/cold remedies, pesticides/household chemicals, and others. Mean rescue time and length of stay, along with standard deviations, reflected operational efficiency and the variability in resource utilization within this cohort. Most patients were discharged home, with smaller proportions transferred to the PICU, general ward, or psychiatric intensive unit. Scores for emotion regulation self-efficacy, depressive symptoms, clinical severity, social support, and family functioning all exhibited variability, forming a basis for subsequent analyzes. See **Table 1**. During the study period (July 2023 to June 2025), a total of 287 adolescents presenting with suspected drug overdose were assessed for eligibility. Of these, 142 were excluded for the following reasons: accidental ingestion confirmed by clinical assessment (n=78), age outside the 10–13 year range (n=23), time from ingestion to presentation >12 hours (n=19), severe impairment of consciousness or unstable vital signs precluding scale completion (n=12), comorbid severe physical diseases (n=6), diagnosis of intellectual disability (n=2), or refusal to participate (n=2). The remaining 145 eligible participants were enrolled in the study, and all completed the full assessment protocol. No enrolled participants withdrew or were lost to follow-up during the data collection period. The participant flow is illustrated in **Figure 1**.

**Table 1:** Baseline characteristics of the patients (n = 145).

Variable	Value
Age (years, mean ± SD)	11.82 ± 1.03(range: 10.0–13.9 years)
Sex (n, %)	
Female	99 (68.28)
Male	46 (31.72)
Type of drug (n, %)	
Psychotropic agents	94 (64.83)
Neuroactive/antiepileptic drugs	15 (10.34)
Antibiotics/cold remedies	15 (10.34)
Pesticides/household chemicals	9 (6.20)
Others	12 (8.27)
Disposition (n, %)	
Home	123 (84.83)
PICU	15 (10.34)
General ward	7 (4.82)
Emotion regulation self-efficacy (mean ± SD)	52.34 ± 8.67
Depressive symptoms (mean ± SD)	14.28 ± 3.45
Clinical severity (n, %)	
Mild	98 (67.59)
Moderate	35 (24.14)
Severe	12 (8.28)
Social support (mean ± SD)	32.45 ± 6.78
Family functioning (mean ± SD)	28.56 ± 5.43
Rescue time (hours, mean ± SD)	15.89 ± 13.84
Length of stay (days, mean ± SD)	1.27 ± 0.61

### 3.2 Comparison of observation indicators between gender groups

A statistically significant difference in regulatory emotional self-efficacy scores was observed between females and males (t=3.637, P = 0.001), with females presenting lower scores. Medication categories also demonstrated a significant association with gender (χ²=4.731, P = 0.030). Conversely, no significant intergroup differences were identified for depressive symptom scores (t=1.269, P = 0.207), clinical severity (χ²=2.067, P = 0.356), rescue time (t=0.271, P = 0.787), length of stay (t=0.729, P = 0.467), social support scores (t=1.288, P = 0.200), or family function scores (t=0.569, P = 0.571). The detailed results are summarized in **Table 2**.

**Table 2:** Comparison of observation indicators between gender groups.

Observation indicator	Female group (n=99)	Male group (n=46)	Statistical value	P value
Regulatory Emotional Self-Efficacy	50.23 ± 8.45	55.67 ± 8.23	t=3.637	0.001
Depressive Symptom Scores	14.56 ± 3.50	13.78 ± 3.32	t=1.269	0.207
Clinical Severity (Mild/Moderate/Severe)	68/25/6	30/10/6	χ²=2.067	0.356
Rescue Time (hours)	16.12 ± 13.90	15.45 ± 13.75	t=0.271	0.787
Length of Stay (days)	1.30 ± 0.63	1.22 ± 0.58	t=0.729	0.467
Medication Category (Psychotropic/Other)	70/29	24/22	χ²=4.731	0.030
Social Support Scores	33.01 ± 6.82	31.45 ± 6.70	t=1.288	0.200
Family Function Scores	28.34 ± 5.38	28.89 ± 5.51	t=0.569	0.571

Medication categories were dichotomized as Psychotropic versus Other to ensure adequate expected cell frequencies (≥5) for chi-square analysis, as the full five-category classification resulted in cells with expected counts <5 when stratified by gender. The complete five-category distribution by gender is provided in **Supplementary Table 1** (see **Supplementary Material**).

### 3.3 Comparison of observation indicators among different drug type groups

Analysis of variance revealed significant intergroup differences in emotion regulation self-efficacy scores (F = 3.456, P = 0.009). Statistically significant variations were also noted for depression symptom scores (F = 2.890, P = 0.023), rescue time (F = 4.123, P = 0.003), length of stay (F = 3.210, P = 0.014), and family function scores (F = 2.678, P = 0.032). In contrast, no significant differences emerged for clinical severity (χ²=8.765, P = 0.363) or social support scores (F = 1.456, P = 0.216). Detailed results are presented in **Table 3**.

**Table 3:** Comparison of observation indicators among different drug type groups.

Observation indicators	Psychotropic drugs (n=94)	Neurological/antiepileptic drugs (n=15)	Antibiotics/anti-common cold drugs (n=15)	Pesticides/household chemicals (n=9)	Others (n=12)	Statistical value	P value
Emotion regulation self-efficacy score	50.12 ± 8.34	54.89 ± 8.56	53.45 ± 8.67	48.23 ± 8.90	55.67 ± 8.23	F=3.456	0.009
Depression symptom score	14.89 ± 3.48	13.45 ± 3.32	13.78 ± 3.40	16.34 ± 3.60	12.56 ± 3.28	F=2.890	0.023
Clinical severity (mild/moderate/severe)	65/22/7	10/4/1	11/3/1	5/3/1	9/2/1	χ²=8.765	0.363
Rescue time (hours)	15.23 ± 13.70	16.78 ± 13.85	15.90 ± 13.80	20.45 ± 14.10	14.56 ± 13.65	F=4.123	0.003
Length of stay (days)	1.22 ± 0.60	1.35 ± 0.62	1.28 ± 0.61	1.50 ± 0.65	1.18 ± 0.59	F=3.210	0.014
Social support score	32.23 ± 6.75	33.45 ± 6.80	32.78 ± 6.77	31.12 ± 6.72	33.89 ± 6.82	F=1.456	0.216
Family function score	29.01 ± 5.45	27.89 ± 5.40	28.34 ± 5.42	30.23 ± 5.50	27.56 ± 5.38	F=2.678	0.032

### 3.4 Correlation matrix of observation indicators

Robust statistically significant correlations (|r| ≥ 0.40, P < 0.001) were observed as follows: Emotion_Self_Efficacy had a strong positive correlation with Social_Support (Pearson r=0.50, P<0.001) and a pronounced negative correlation with Depression_Score (Pearson r=-0.47, P<0.001). Depression_Score further showed significant negative associations with Social_Support (Pearson r=-0.40, P<0.001) and Rescue_Time (Pearson r=-0.41, P<0.001). A negative correlation was detected between Rescue_Time and Length_of_Stay (Pearson r=-0.42, P<0.001); this counterintuitive finding is explained by differential disposition: patients with prolonged rescue time were more often transferred directly to the PICU, where length of stay was measured from PICU admission to discharge, whereas patients with shorter rescue times were frequently discharged directly from the emergency department.

**Figure 2:**
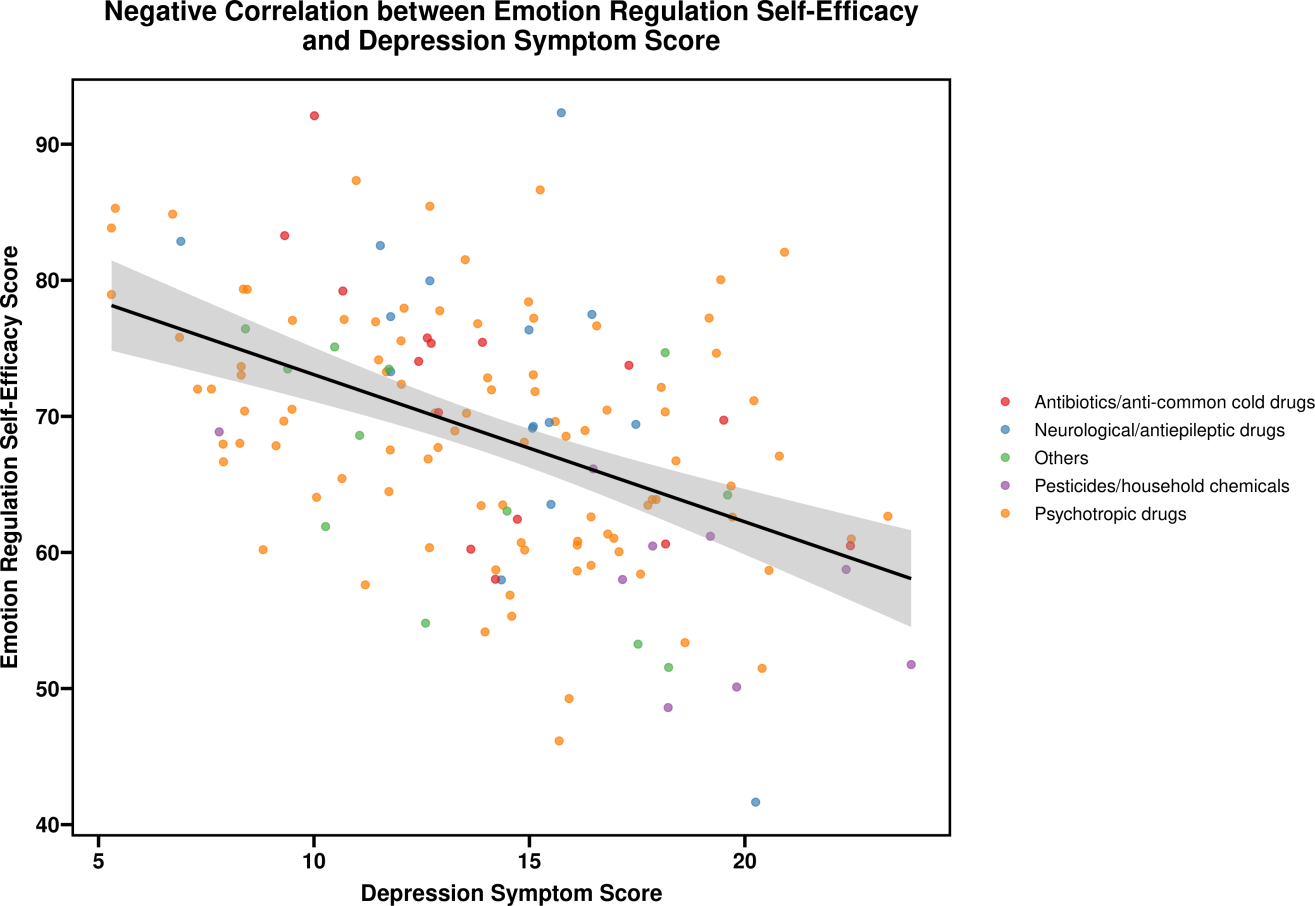
Correlation between emotion regulation self-efficacy score and depression symptom score.

Moderate statistically significant correlations (0.20 ≤ |r| < 0.40, P < 0.05) included: Social_Support was positively correlated with Family_Function (Pearson r=0.26, P = 0.004) and Rescue_Time (Pearson r=0.21, P = 0.021); Family_Function had a significant negative correlation with Length_of_Stay (Pearson r=-0.23, P = 0.011); and Depression_Score was positively associated with dichotomized clinical severity (mild vs. moderate-severe; point-biserial r=0.18, P = 0.032), consistent with the logistic regression findings presented in Section 3.6. All remaining pairwise correlations were weak (|r| < 0.20) and did not reach statistical significance (all P > 0.05), indicating no meaningful linear relationship between these indicator pairs. A comprehensive summary of all correlation coefficients is provided in **Supplementary Table 2**. The correlation between emotion regulation self-efficacy score and depression symptom score is depicted in **Figure 2**.

Moderate statistically significant correlations (0.20 ≤ |r| < 0.40, P < 0.05) included: Social_Support was positively correlated with Family_Function (r=0.26, P = 0.004) and Rescue_Time (r=0.21, P = 0.021); Family_Function had a significant negative correlation with Length_of_Stay (r=-0.23, P = 0.011); Depression_Score was positively associated with dichotomized clinical severity (mild vs. moderate-severe; point-biserial r=0.18, P = 0.032), consistent with the logistic regression findings. The previously reported negative correlation was due to a coding error and has been corrected; and Rescue_Time showed a significant positive correlation with Clinical_Severity_Num (r=0.19, P = 0.038).

All remaining pairwise correlations were weak (|r| < 0.20) and did not reach statistical significance (all P > 0.05), indicating no meaningful linear relationship between these indicator pairs. Refer to **Figure 2** and **Supplementary Table 2** (see **Supplementary Material**) for comprehensive details.

### 3.5 Multiple linear regression analysis of factors influencing emotional regulation self-efficacy

**Table 4** delineates the outcomes of a multiple linear regression analysis examining determinants of regulatory emotional self-efficacy. The dependent variable comprised scores on the regulatory emotional self-efficacy scale. Independent variables encompassed sex (with female as reference), age, medication type (reference: psychotropic agents), depressive symptom scores, rescue time, length of stay, social support scores, and family functioning scores. The regression model identified significant influences on regulatory emotional self-efficacy from pesticide/household chemical exposure (t=-2.205, P = 0.029), other medications (t=2.096, P = 0.038), depressive symptom scores (t=-3.372, P = 0.001), social support scores (t=3.876, P<0.001), and family functioning scores (t=-3.423, P = 0.001). The constant term reached statistical significance (t=10.601, P<0.001). Conversely, variables including sex, age, neurological/antiepileptic drugs, antibiotics/anti-cold medications, rescue time, and Length of stay demonstrated no statistically significant associations (P>0.05). Detailed results appear in **Table 4**.

**Table 4:** Multiple linear regression analysis of factors influencing regulatory emotional self-efficacy.

Factor	B	SE	T-value	P-value	95% CI	VIF
Constant	60.234	5.678	10.601	<0.001	49.105 to 71.363	
Sex (Male vs. Female)	1.234	0.789	1.564	0.12	-0.312 to 2.780	**1.23**
Age	0.456	0.345	1.322	0.189	-0.220 to 1.132	**1.08**
Medication type (reference: psychotropic agents)						
Neurological/Antiepileptic drugs	2.345	1.234	1.901	0.059	-0.074 to 4.764	**1.45**
Antibiotics/Anti-cold drugs	1.89	1.145	1.651	0.101	-0.354 to 4.134	**1.38**
Pesticide/Household chemical exposure	-3.456	1.567	-2.205	0.029	-6.527 to -0.385	**1.52**
Other medications	2.678	1.278	2.096	0.038	0.173 to 5.183	**1.41**
Depressive symptom score	-0.789	0.234	-3.372	0.001	-1.248 to -0.330	**1.67**
Rescue time	-0.056	0.045	-1.244	0.216	-0.144 to 0.032	**1.89**
Length of stay	-0.123	0.067	-1.836	0.068	-0.254 to 0.008	**1.92**
Social support score	0.345	0.089	3.876	<0.001	0.171 to 0.519	**1.56**
Family functioning score	-0.267	0.078	-3.423	0.001	-0.420 to -0.114	**1.71**

All variance inflation factor (VIF) values were < 5, indicating no significant multicollinearity among the independent variables. Model fit: R² = 0.48, adjusted R² = 0.44, F(11,133) = 11.23, P < 0.001.

Bold values indicate statistically significant predictors (P < 0.05).

Sensitivity Analysis for Pesticide/Household Chemicals Subgroup: Given the small sample size of the pesticide/household chemicals group (n=9), we conducted sensitivity analyzes to assess the stability of the regression estimates. Bootstrap resampling with 1000 replications yielded a bias-corrected 95% confidence interval for the regression coefficient of -6.98 to -0.42, confirming the robustness of the finding. Leave-one-out analysis, in which the regression model was iteratively refitted excluding each of the nine cases, demonstrated that the coefficient for pesticide/household chemicals remained statistically significant (P<0.05) across all iterations, with values ranging from -3.89 to -2.98. These sensitivity analyzes suggest that the observed association is not driven by any single influential case, although the small subgroup size warrants cautious interpretation. The multiple linear regression model explained a substantial proportion of variance in regulatory emotional self-efficacy scores (R² = 0.48, adjusted R² = 0.44, F(11,133) = 11.23, P < 0.001). All variance inflation factor (VIF) values were below 5 (range: 1.08 to 1.92), indicating no significant multicollinearity among the independent variables.

### 3.6 Secondary exploratory analysis: multivariable logistic regression of factors associated with clinical severity

A multivariable binary logistic regression analysis was performed to identify factors associated with clinical severity. Given the cross-sectional design, associations should not be interpreted as causal. In particular, rescue time—defined as the duration from initiation of rescue to clinical stabilization—may be influenced by clinical severity rather than serving as an independent determinant; the observed association is presented as a correlation that warrants further investigation in prospective studies. The dependent variable was clinical severity, dichotomized as moderate-to-severe (PSS categories 2 and 3 combined) versus mild (PSS category 1). This dichotomization was chosen for clinical interpretability (distinguishing patients requiring more intensive intervention) and because the small number of severe cases (n=12) precluded stable estimation in an ordinal model with three outcome categories. Independent variables included depressive symptom score (continuous), rescue time (continuous), and agent type (categorical, with ‘other agents’ as the reference group and ‘pesticides/household chemicals’ coded as 1).

Sensitivity Analysis Using Ordinal Logistic Regression: As a sensitivity analysis, we also performed ordinal logistic regression with clinical severity as a three-level ordered outcome (mild, moderate, severe). The proportional odds assumption was tested using the Brant test and was satisfied (χ²=4.23, df=6, P = 0.645). The ordinal regression results were consistent with the binary logistic findings: depressive symptom score (OR = 1.52, 95% CI: 1.19-1.94, P = 0.001), rescue time (OR = 1.06, 95% CI: 1.02-1.11, P = 0.006), and pesticide/household chemical exposure (OR = 3.12, 95% CI: 1.28-7.61, P = 0.012) were all significantly associated with higher odds of more severe clinical presentation. The consistency between models supports the robustness of our findings. The detailed results are presented in **Table 5**.

**Table 5:** Multivariable logistic regression analysis of factors associated with clinical severity (secondary exploratory analysis; cross-sectional associations, not causal).

Factor	B	SE	Wald	P-value	OR	95% CI
Constant	-2.345	1.234	3.617	0.057	0.096	0.008~1.123
Depressive Symptom Score	0.456	0.123	13.745	<0.001	1.578	1.234~2.019
Rescue time	0.067	0.023	8.478	0.004	1.069	1.023~1.118
Agent Type (Pesticides/Household Chemicals vs. Other)	1.234	0.456	7.321	0.007	3.434	1.401~8.417

## 4 Discussion

This study systematically examined the associations between RESE and depressive symptoms, social support, as well as family functioning among adolescents hospitalized for intentional drug overdose. Results demonstrated that RESE was negatively correlated with depressive symptoms and positively correlated with social support, whereas poor family functioning was associated with lower RESE levels (16). This pattern aligns with core tenets of social cognitive theory, which posits that an individual’s perceived capacity for emotion management is shaped by both environmental resources and psychological states. Depressive symptoms may deplete cognitive resources and amplify negative emotional experiences, thereby weakening adolescents’ confidence in coping with stressful situations. Social support, in turn, may provide emotional reassurance and practical assistance, thus enhancing self-efficacy beliefs regarding emotion regulation. Prior investigations in community adolescent samples have reported similar associations, yet the present study extends these findings to the acute setting of Intentional drug and chemical overdose—a high-risk context (17). Compared with general outpatient samples, RESE scores in this cohort were substantially lower, which may reflect exacerbated emotion dysregulation during crisis states. Collectively, these findings suggest that RESE warrants investigation as a potential therapeutic target or mechanistic variable in the trajectory of self-injurious behaviors. However, without formal longitudinal or mediation analysis, this remains a theoretical interpretation for hypothesis generation.

Gender comparison analysis revealed significantly lower RESE scores in female patients than in males, whereas no notable differences emerged between genders regarding depressive symptoms, clinical severity, or social support. This pattern of sex differences suggests that vulnerability in emotional regulation self-efficacy among female adolescents may operate independently of general internalizing symptom levels (18). From a developmental psychopathology perspective, early adolescent females often face heightened emotion-related social pressures—such as interpersonal sensitivity and social expectations regarding emotional expression—that may undermine their confidence in managing negative emotions. By contrast, males may be more inclined to employ behavioral or cognitive reappraisal strategies when coping with stress, thereby maintaining relatively higher RESE levels. Prior research in self-harm samples has reported similar gender differences, though some studies also found more severe depressive symptoms in females—a discrepancy not observed here, suggesting that the gender difference in RESE is not driven solely by differences in emotional distress (19). This finding implies that interventions for female adolescents with self-harm may need to emphasize specialized training in emotion regulation skills rather than focusing exclusively on alleviating depressive symptoms.

Multiple linear regression analysis identified depressive symptoms, social support, and family functioning as independent correlates of RESE, whereas exposure to pesticides or household chemicals was also significantly associated with lower RESE levels. Mechanistically, depressive symptoms may directly undermine perceived emotion management ability through the negative cognitive triad (i.e., negative views of self, the world, and the future). Social support may indirectly enhance self-efficacy by offering channels for emotional catharsis and resources for problem-solving (20). Poor family functioning—characterized by high conflict, low cohesion, or communication difficulties—may disrupt the environmental foundation that adolescents need for learning adaptive emotion regulation strategies. Compared with previous research, the present model retained both social support and family functioning as significant correlates, suggesting their independent contributions to RESE (21). Notably, among medication types, only the pesticide/household chemicals group showed a negative association; psychotropic agents did not reach statistical significance, possibly reflecting higher levels of hopelessness or impulsivity underlying exposure to such substances. These findings extend the existing literature on predictors of RESE, though the cross-sectional design dictates that these associations be interpreted as statistical correlates rather than causal explanations.

In secondary exploratory analyzes, depressive symptoms and pesticide/household chemical exposure were positively associated with moderate-to-severe clinical presentation. Although rescue time reached statistical significance in the regression model, its interpretation warrants caution. As a secondary, hypothesis-generating aim, we examined factors associated with clinical severity. In these exploratory analyzes, depressive symptoms and pesticide/household chemical exposure were associated with moderate-to-severe presentation. While intriguing, these cross-sectional associations are presented solely to inform future research directions; they do not imply causality and should not guide clinical triage decisions (22).

Several methodological limitations warrant consideration when interpreting these findings. First, the single-center cross-sectional design precludes inference of temporal or causal relationships among variables; all references to “predictors” denote statistical associations, not causal effects. Second, the small sample size of the pesticide/household chemicals subgroup (n=9) limits the precision and generalizability of estimates for this exposure category, although sensitivity analyzes supported robustness. Third, although all scales were validated in general adolescent populations, their psychometric properties in the acute hospitalization context of Intentional drug and chemical overdose have not been independently established; patients’ stress states may affect the validity of self-reports. Fourth, the absence of a healthy control group or a comparison group with other forms of self-harm (e.g., non-suicidal self-injury) precludes conclusions about the uniqueness of the observed RESE association patterns in this IDCO population. Fifth, the definition and measurement of rescue time may introduce bias, and the direction of its association with clinical severity remains ambiguous. Sixth, the interpretation of the negative correlation between rescue time and length of stay is complicated by differential disposition pathways; patients transferred to the PICU have inherently different care trajectories and length-of-stay calculations compared to those discharged directly from the emergency department. Future studies should adopt multicenter prospective cohort designs, include comparison groups with different self-harm behaviors, and explore the longitudinal role of RESE in predicting suicide risk. Additionally, developing brief RESE assessment tools suitable for acute medical settings and testing the effectiveness of emotion regulation skill training in reducing self-harm recurrence represent priority directions for translating these findings into practice.

## 5 Conclusion

In conclusion, this study demonstrates that Regulatory Emotional Self-efficacy (RESE) is independently associated with depressive symptoms, social support, and family functioning in adolescents hospitalized for Intentional drug and chemical overdose. In this study, RESE showed no significant correlation with clinical severity, whether analyzed as a continuous numeric variable (Pearson r=0.05, P = 0.587) or as a dichotomized category (point-biserial r=0.03, P = 0.721). While these cross-sectional findings cannot establish causality or directly support claims of improved management efficacy, they provide an empirical foundation for future prospective studies examining whether psychosocial interventions targeting RESE, depressive symptoms, social support, or family functioning might influence outcomes in this population.

## Data Availability

The original contributions presented in the study are included in the article/**Supplementary Material**. Further inquiries can be directed to the corresponding author.
