# Supplementary material for: Factors associated with emotional regulation self-efficacy in adolescents hospitalized for intentional drug and chemical overdose: a cross-sectional study
Source: Front Psychiatry. 2026 May 22;17:1793066. doi: 10.3389/fpsyt.2026.1793066 (PMC13236878; doi:10.3389/fpsyt.2026.1793066)
Supplement: Supplementary file 1 [file Table1.docx]

Supplementary Table S1:

| Medication Category | Female (n=99) | Male (n=46) | Total (N=145) |
| --- | --- | --- | --- |
| Psychotropic agents | 70 (70.7%) | 24 (52.2%) | 94 (64.8%) |
| Neurological/antiepileptic | 9 (9.1%) | 6 (13.0%) | 15 (10.3%) |
| Antibiotics/cold remedies | 8 (8.1%) | 7 (15.2%) | 15 (10.3%) |
| Pesticides/household chemicals | 6 (6.1%) | 3 (6.5%) | 9 (6.2%) |
| Others | 6 (6.1%) | 6 (13.0%) | 12 (8.3%) |
| Chi-square for full table: χ²=5.546, df=4, P=0.236 | | | |
