# Supplementary material for: Factors associated with emotional regulation self-efficacy in adolescents hospitalized for intentional drug and chemical overdose: a cross-sectional study
Source: Front Psychiatry. 2026 May 22;17:1793066. doi: 10.3389/fpsyt.2026.1793066 (PMC13236878; doi:10.3389/fpsyt.2026.1793066)
Supplement: Supplementary file 2 [file Table2.docx]

Supplementary Table S2

| **Indicator** | **1. Emotion_Self_Efficacy** | **2. Depression_Score** | **3. Rescue_Time** | **4. Length_of_Stay** | **5. Social_Support** | **6. Family_Function** | **7. Clinical_Severity_Num** |
| --- | --- | --- | --- | --- | --- | --- | --- |
| **1. Emotion_Self_Efficacy** | **1** | **-0.47 (<0.001)** | **0.12 (0.191)** | **0.04 (0.664)** | **0.50 (<0.001)** | **0.02 (0.828)** | **0.05 (0.587)** |
| **2. Depression_Score** | **-0.47 (<0.001)** | **1** | **-0.41 (<0.001)** | **0.01 (0.914)** | **-0.40 (<0.001)** | **-0.15 (0.102)** | **-0.20 (0.028)** |
| **3. Rescue_Time** | **0.12 (0.191)** | **-0.41 (<0.001)** | **1** | **-0.42 (<0.001)** | **0.21 (0.021)** | **0.14 (0.127)** | **0.19 (0.038)** |
| **4. Length_of_Stay** | **0.04 (0.664)** | **0.01 (0.914)** | **-0.42 (<0.001)** | **1** | **0.01 (0.914)** | **-0.23 (0.011)** | **0.05 (0.587)** |
| **5. Social_Support** | **0.50 (<0.001)** | **-0.40 (<0.001)** | **0.21 (0.021)** | **0.01 (0.914)** | **1** | **0.26 (0.004)** | **0.15 (0.102)** |
| **6. Family_Function** | **0.02 (0.828)** | **-0.15 (0.102)** | **0.14 (0.127)** | **-0.23 (0.011)** | **0.26 (0.004)** | **1** | **0.08 (0.385)** |
| **7. Clinical_Severity_Num** | **0.05 (0.587)** | **-0.20 (0.028)** | **0.19 (0.038)** | **0.05 (0.587)** | **0.15 (0.102)** | **0.08 (0.385)** | **1** |

NOTE:*The negative correlation between Rescue_Time and Length_of_Stay (r=-0.42) reflects differential measurement and disposition patterns rather than a true inverse clinical relationship; see main text Discussion for details.
